# Myostatin: a potential therapeutic target for metabolic syndrome

**DOI:** 10.3389/fendo.2023.1181913

**Published:** 2023-05-23

**Authors:** Ming Yang, Chongbin Liu, Na Jiang, Yan Liu, Shilu Luo, Chenrui Li, Hao Zhao, Yachun Han, Wei Chen, Li Li, Li Xiao, Lin Sun

**Affiliations:** ^1^ Department of Nephrology, The Second Xiangya Hospital of Central South University, Changsha, China; ^2^ Hunan Key Laboratory of Kidney Disease and Blood Purification, Changsha, Hunan, China

**Keywords:** myostatin, metabolic syndrome, obesity, diabetes, lipid

## Abstract

Metabolic syndrome is a complex metabolic disorder, its main clinical manifestations are obesity, hyperglycemia, hypertension and hyperlipidemia. Although metabolic syndrome has been the focus of research in recent decades, it has been proposed that the occurrence and development of metabolic syndrome is related to pathophysiological processes such as insulin resistance, adipose tissue dysfunction and chronic inflammation, but there is still a lack of favorable clinical prevention and treatment measures for metabolic syndrome. Multiple studies have shown that myostatin (MSTN), a member of the TGF-β family, is involved in the development and development of obesity, hyperlipidemia, diabetes, and hypertension (clinical manifestations of metabolic syndrome), and thus may be a potential therapeutic target for metabolic syndrome. In this review, we describe the transcriptional regulation and receptor binding pathway of MSTN, then introduce the role of MSTN in regulating mitochondrial function and autophagy, review the research progress of MSTN in metabolic syndrome. Finally summarize some MSTN inhibitors under clinical trial and proposed the use of MSTN inhibitor as a potential target for the treatment of metabolic syndrome.

## 1 Brief review of MSTN

Myostatin (MSTN), a member of TGF-β family, also known as growth differentiation factor 8 (GDF8), is a potent inhibitor of skeletal muscle development (1–3). It was first identified by McPherron et al. in 1997 and it was found MSTN is exclusively expressed in the myotome compartment of developing somites in the early stages of embryogenesis (4). Targeting mutant MSTN resulted in large and extensive increases in skeletal muscle mass in mice, with the muscle mass of mutant animals being 2-3 times heavier than that of wild-type animals (4). This suggests that MSTN is a negative regulator of skeletal muscle growth.

Early studies suggested that it is mainly expressed in skeletal muscle. However, with the deepening of research on it, it is also expressed in tissues other than skeletal muscle, such as adipose tissue (5), kidney (6) and heart (7). Before being secreted, its precursor is synthesized, which consists of an N-terminal signal sequence, an N-prodomain region, and a biologically active C-terminal domain (8, 9). The precursor needs to be cleaved twice to generate active MSTN. The first is the removal of a 24-amino acid signal peptide by Furin family enzymes (10), and the second is the cleavage by bone morphogenetic protein 1 (BMP-1)/Tolloid matrix metalloproteinases to generate mature myostatin dimers (11). This allows the active myostatin ligand to dissociate from the inhibitory N-terminal propeptide domain, allowing interaction with the receptor (12, 13).

**Figure 1:**
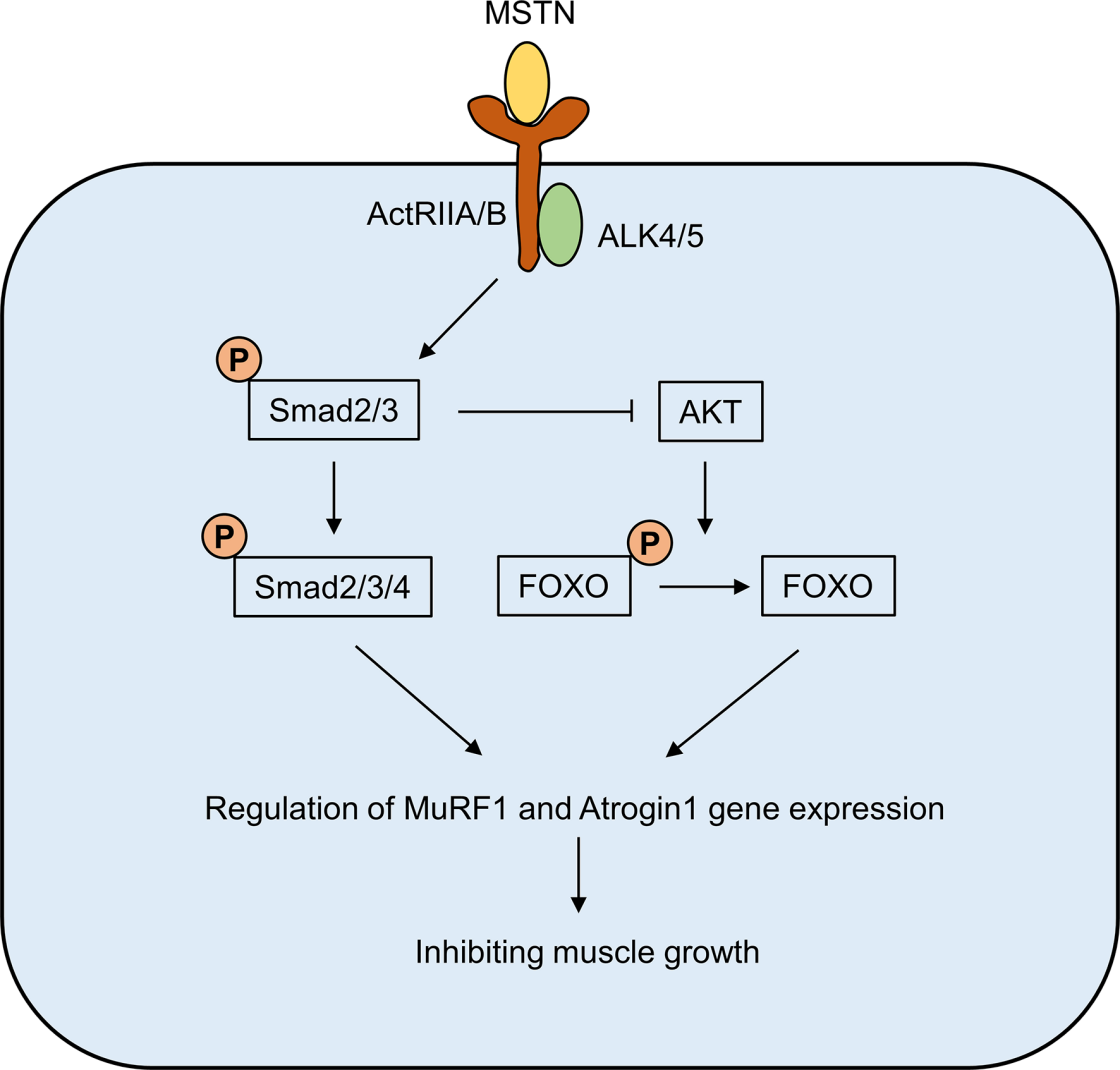
The signaling pathway of MSTN. The binding of MSTN to ActRIIA and ActRIIB requires the involvement of activin-like kinase (ALK) 4/5, which subsequently leads to the phosphorylation of the downstream Smad2/3 complex, which in turn recruits Smad4. Meanwhile, phosphorylated Smad2/3 also inhibited AKT activation and promoted the dephosphorylated of FOXO. The Smad complex and dephosphorylated FOXO can enter the nucleus and act as transcription factors to regulate the expression of MuRF1 and Atrogin1 muscle atrophy-related proteins.

MSTN is mostly present in the circulation as a bound inactive form (14, 15). Two activin type II receptors, ActRIIA and ActRIIB, are receptors that mediate the physiological effects of MSTN. The binding of MSTN to ActRIIA and ActRIIB requires the involvement of activin-like kinase (ALK) 4/5, which subsequently leads to the phosphorylation of the downstream Smad2/3 complex, which in turn recruits Smad4 (16, 17). Meanwhile, phosphorylated Smad2/3 also inhibited AKT activation and promoted the dephosphorylated of FOXO (18). The Smad complex and dephosphorylated FOXO can enter the nucleus and act as transcription factors to regulate the expression of MuRF1 and Atrogin1 muscle atrophy-related proteins. MuRF1 and Atrogin1 could promote ubiquitination of muscle proteins, thereby accelerating their degradation through the proteasome pathway and ultimately inhibiting muscle growth (**Figure 1**) (19, 20).

In addition to the classical Smad signaling pathway, MSTN has also been reported to regulate cell growth by activating the c-Jun N-terminal kinase (JNK) signaling pathway. When ActRIIB was knocked out, MSTN-mediated JNK activation was significantly reduced and further intervention with JNK specific inhibitor SP600125 notably inhibited the MSTN-induced p21 up-regulation and differentiation marker gene expression down-regulation (21). In addition, MSTN has also been reported to be involved in the regulation of cell growth possibly through extracellular signal-regulated kinase (ERKs), p38 mitogen-activated protein kinases and Wnt signaling pathway (22–24).

## 2 The regulation of MSTN expression

Many proteins/compounds are involved in the regulation of MSTN expression. IGF-1 is a growth factor that plays a crucial role in regulating cell proliferation and it has also been reported to be involved in the process of transcriptional regulation of MSTN. Yang et al. have shown that IGF-1 intervention could upregulate myostatin expression through the phosphatidylinositol 3-kinase pathway (25). Further research showed that IGF-1 could activate the Ca^2+^-dependent nuclear factor of activated T cells (NFAT) transcription factor to bind to the MSTN promoter through the phospholipase C gamma (PLCγ)/inositol 1,4,5-triphosphate (IP3) signaling pathways, thereby regulating the transcription of MSTN (26).

In addition, a CCAAT box and a C/EBP-binding element (CBE) in the MSTN promoter are the two potential C/EBP-binding sequences (27). When treated with glucocorticoids, the activity of C/EBP-δ promoter was notably up-regulated after 1 hour of dexamethasone treatment, while the activity MSTN promoter was not significantly increased until 24 hours or more of intervention (27). Furthermore, by cotransfection with the C/EBP-δ expression vector, the myostatin promoter-reporter construct showed a significant increase in activity. Mutation of the CCAAT box in the MSTN promoter partially blocked the MSTN promoter activity, whereas mutation of the CBE completely abrogated glucocorticoid regulation of the MSTN promoter activity (27). Moreover, FOXO1 has also been reported to be a transcription factor of MSTN, and FOXO1 could increase myostatin mRNA levels and up-regulate myostatin promoter activity, and the mutations in the binding site to FOXO1 in the MSTN promoter significantly reduced the activity of the MSTN promoter (28). Moreover, The NF-κB p65 subunit can also bind to the myostatin promoter to stimulate MSTN gene transcription (29). In order to realize MSTN as a target for disease treatment, its specific agonists or inhibitors need to be identified, which will be described in detail in the following part.

## 3 MSTN in regulating cell metabolism

### 3.1 Mitochondria

Mitochondria are energy factories in cells, synthesizing large amounts of ATP to provide energy for the body’s activities (30–32). In the case of metabolic disorder, the morphology, number and function of mitochondria in tissues will change. On the one hand, damaged and abnormal mitochondria can be cleared through a process known as mitophagy, and when damaged mitochondria exceed their own clearance, mitochondrial contents are released into the cell, leading to oxidative stress, inflammation and apoptosis (33–35). Therefore, maintaining the stability of mitochondrial function is an important way to prevent disease progression. Interestingly, studies have also reported that MSTN is closely related to mitochondrial function.

Ploquin et al. have demonstrated that when MSTN was knocked out in mice, the respiratory coupling of mitochondria was reduced in intermyofibrillar, and basal oxygen consumption was significantly increased, lipid peroxidation levels were significantly reduced and the antioxidant glutathione system was notably upregulated (36). Moreover, when mice lack MSTN, the rate of ATP production from oxidative phosphorylation (OXPHOS) is inhibited in skeletal muscle, and the activity of respiratory chain complex is also decreased (37). The molecular mechanism may be that Smad2/3 is insufficiently bound to the promoter region of Idh2 and Idh3a (key rate-limiting enzymes associated with the TCA cycle), thus affecting OXPHOS of the cell (37). Similar result was also observed that MSTN knockout may inhibit mitochondrial function by inhibiting AMPK/SIRT1/PGC1α signaling pathway (38).

Furthermore, MSTN may also regulate mitochondrial function by affecting lipid metabolism. Compared with the control group, the expression levels of lipid membrane transporters (CD36, FABP3, FATP1, and FATP4) and proteins related to lipid oxidation pathways were significantly decreased in the muscle of MSTN-deficient mice. Further analysis of the composition differences of phospholipids and fatty acids in mitochondrial membrane by chromatography showed that the ratio of mitochondrial cardiolipin proportion of MSTN knockout mice was reduced compared with other phospholipids (39). This suggests that MSTN may affect mitochondrial function by regulating lipid composition in mitochondrial membrane. Interestingly, the abnormal cardiolipin content in muscle mitochondrial membrane caused by MSTN deficiency can be corrected by endurance training (40).

### 3.2 Autophagy

Autophagy is a process of maintaining cellular homeostasis through lysosome degradation of cellular damage or excessive organelles, proteins and other endogenous substances (41–43). Abnormalities in autophagy are closely related to the occurrence and development of a variety of metabolic diseases such as obesity, diabetes and non-alcoholic fatty liver disease (NAFLD) (44–46). Interestingly, MSTN has also been reported to be involved in the regulation of autophagy homeostasis in cells. The decreased of basal autophagy flux and ATP content were observed in muscle of MSTN knockout mice (47), while the treatment of MSTN can increase the expression of autophagy associated protein LC3II and autophagosome formation in skeletal muscle (48) and the increased autophagic flux induced by MSTN was blocked by elevating levels of G protein-coupled receptor kinase 2 (GRK2) (49). Furthermore, Anand et al. have shown that the mRNA expressions of MSTN and BECN1 (autophagy associated proteins) were increased in skeletal muscle of patients with sarcopenia compared with controls (50). These results imply that MSTN inhibits muscle hypertrophy by enhancing autophagy levels in skeletal muscle cells. However, MSTN regulates autophagy differently in cells with myocardial hypertrophy. There is an over-activated autophagy level in myocardial cells during myocardial hypertrophy, while in mice lacking MSTN, abdominal aortic coarctation (AAC) was aggravated and accompanied by an increase in angiotensin II-induced autophagy, which was reversed *in vivo* and *in vitro* by MSTN treatment (51). Mechanically, MSTN-mediated anti-autophagy mediated by inhibition of AMPK/mTOR and activation of the PPARγ/NF-κB signaling pathway (51). This suggests that MSTN regulates autophagy in different tissues and diseases in different ways. However, whether MSTN has an effect on autophagy in tissues other than skeletal and cardiac muscles needs to be observed in future studies.

## 4 MSTN and metabolic syndrome

### 4.1 MSTN in obesity and hyperlipemia

As a manifestation of metabolic disorder, obesity has been shown to be associated with increased myostatin expression. The mRNA levels of MSTN and its receptor ActRIIb were increased by more than 50-to 100-fold in subcutaneous and visceral fat of ob/ob mice (a mouse model of obesity) compared with wild-type mice (52). Similarly, the concentrations of myostatin were increased in obese nondiabetic subjects compared with lean subjects and were positively correlated with the insulin resistance index and negatively correlated with the insulin sensitivity index (53). Moreover, high-fat feeding could significantly increase the body weight and the expression of MSTN in muscle of high-fat diet induced obesity susceptible mice models, but the expression of MSTN in muscle of high-fat diet induced obesity resistant mice does not change significantly (54). These studies imply that MSTN may play a key role in obesity, and subsequent studies have demonstrated that altering MSTN expression can affect the development of obesity.

**Figure 2:**
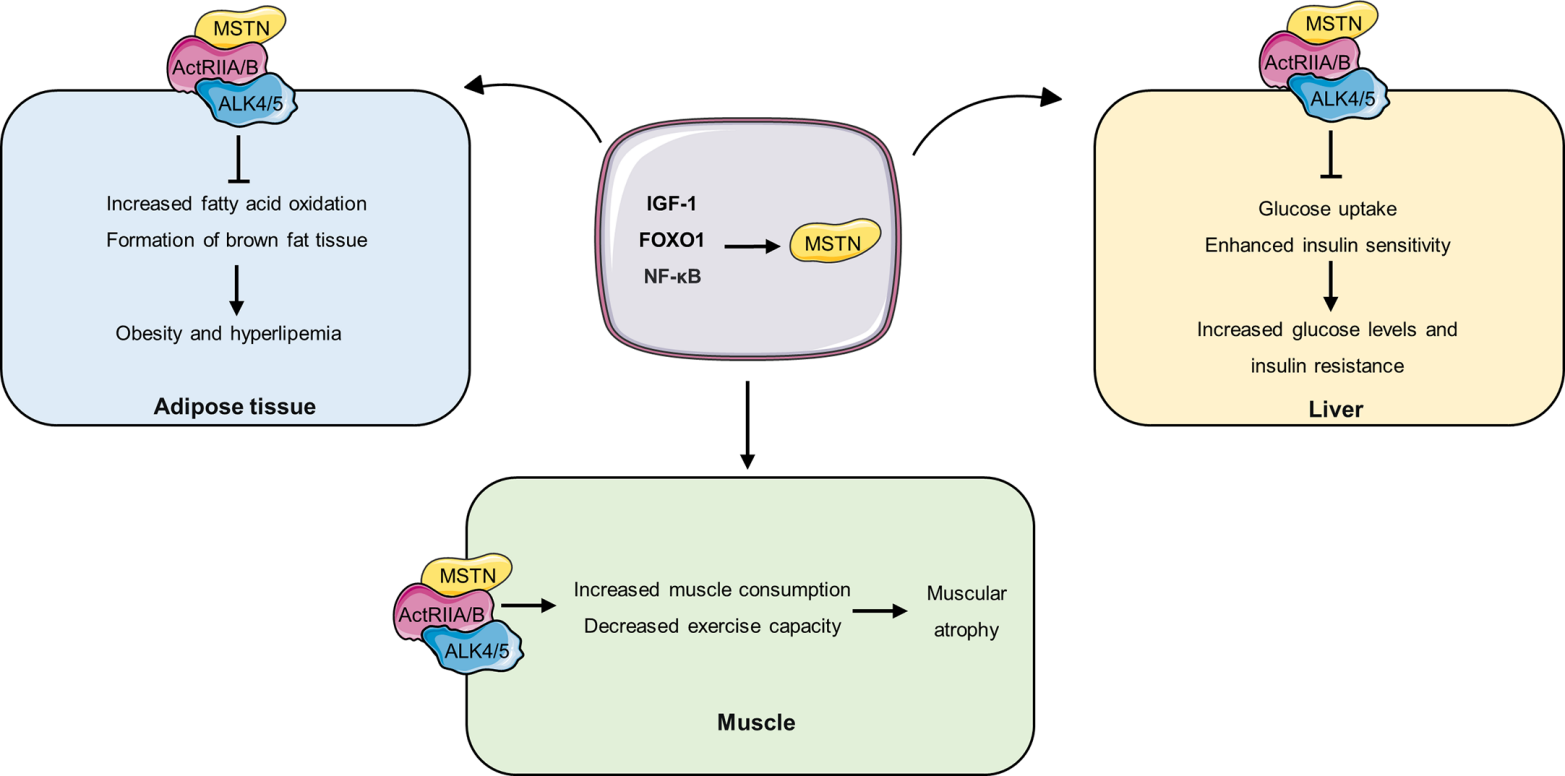
The role of MSTN in different tissues. IGF-1, FOXO1 and NF promote the expression and secretion of MSTN, and MSTN circulates to different tissues and binds to ActRIIA/B receptors on the cell. In adipose tissue, MSTN inhibits fatty acid oxidation and brown adipose tissue formation, thereby promoting obesity and hyperlipidemia. In the liver, MSTN inhibits hepatic glucose uptake and decreases insulin sensitivity, thereby increasing blood glucose levels and insulin resistance. In muscle, MSTN accelerates muscle consumption and decreased exercise capacity, resulting in muscle atrophy.

There were decreased muscle mass, decreased myocardial mass, and increased epididymal fat mass were observed in MSTN overexpressing mice (55). Similar result was also observed that the depletion of MSTN could reduce the age-related adipose tissue mass increase and partially reduce the obesity and diabetic phenotypes in agouti lethal yellow (A^y^) and obese (Lep^ob/ob^) mouse models of obesity and diabetes (56). In addition, Zhang et al. also demonstrated that the absence of MSTN induces reduced fat accumulation in mice fed a high-calorie diet (57). Mechanistically, loss of MSTN mediates fat accumulation suppression through two independent mechanisms. On the one hand, deficiency of MSTN upregulates enzymes involved in lipolysis and mitochondrial fatty acid oxidation (e.g., CPT1a and CPT2), thereby increasing fatty acid oxidation in peripheral tissues and reducing lipid accumulation. On the other hand, its absence also promoted the formation of brown fat in white adipose tissue in mice (white adipose tissue is an energy storage organ, while brown fat is rich in mitochondria and is involved in thermogenesis by consuming fat) (**Figure 2**) (57). Furthermore, intervention with the MSTN antagonist sActRIIB in wild-type HFD-fed mice significantly reduced obesity in mice (57). Moreover, compared with wild-type HFD-fed mice, HFD-fed male MSTN-deficient mice had reduced plasma cholesterol and triglyceride levels and reduced plasma TNF-α levels by approximately 40 percent, and reduced insulin resistance (58). These results suggest that anti-MSTN therapy may be a potential target for delaying obesity and hyperlipemia. However, there is still disagreement about the role of MSTN in obesity. Zhu et al. have shown that the content of lipids in 3T3-L1 preadipocytes treated with myostatin significantly decreased compared with untreated cells (59). This difference may be due to different cell lines’ different responses to MSTN. However, in future studies, the molecular mechanism of MSTN in obesity needs to be further revealed, which is conducive to the development of anti-obesity drugs targeting MSTN.

### 4.2 MSTN in diabetes

The relationship between diabetes mellitus and MSTN has also been partially revealed. The concentration of MSTN is closely related to the occurrence and development of diabetes or insulin resistance. Dial et al. shown that serum myostatin levels were significantly higher in type 1 diabetes (T1D) patients than in the control group and were higher in T1D women than in T1D men (60). Similar results have been observed in type 2 diabetes (T2D) that the mRNA levels of MSTN were higher in the muscles of patients with T2D than in the control group and MSTN mRNA was correlated with homeostasis model assessment of insulin resistance (HOMA2-IR) and plasma IL-6 level (61). Similar results have been observed that compared with the control group, mRNA expression of MSTN was increased in muscle and subcutaneous adipose tissue of diabetic rats, and mRNA expression of MSTN receptor (ActRIIB) was increased in brown adipose tissue (62). Moreover, Hittel et al. found a strong correlation between plasma MSTN levels and insulin sensitivity, and that injection of myostatin induced insulin resistance in mice (63).

Interestingly, inhibiting the level of MSTN could relieve insulin resistance and diabetes. The elderly men with T2D had significantly higher circulating MSTN concentration and lower muscle strength, while RT training can significantly reduce circulating MSTN levels and increase muscle strength (64). The increased MSTN expression was detected in in skeletal muscle of type 2 diabetic KKAy mice or palmitate treated C2C12 cells, while astragalus polysaccharide could downregulated the expression of MSTN and improve insulin sensitivity (65). By crossing lipodystrophy mice (A-ZIP/F1) with mice expressing a dominant negative MSTN receptor (activin receptor type IIB) in muscle, inhibition of MSTN action in A-ZIP/F1 mice notably lower the levels of blood glucose, serum insulin, triglyceride, and triglyceride synthesis rate, and enhanced insulin sensitivity (66). Moreover, by crossing Akita diabetic mice with myostatin knockout mice, the resulting diabetic myostatin knockout mice had upregulated Glut1 and Glut4 proteins and increased glucose uptake capacity, which in turn resulted in significantly down-regulated resting blood glucose levels and significantly reduced associated diabetes symptoms (67). Similarly, the intervention of MSTN significantly reduced basal and insulin-induced phosphorylation of IRS-1 tyrosine (Tyr495), as well as PI3K expression and activation (68). In addition, MSTN also inhibited AMPK activation and down-regulated Glut4 protein expression, which impaired systemic glucose homeostasis (68). Similar result was observed that myostatin significantly inhibits insulin-stimulated glucose uptake and Akt phosphorylation in hepatocytes (69). The changes of Mss51 were most pronounced in the muscle transcription profiles of MSTN-knockout mice or mice treated with a myostatin/activin inhibitor (ActRIIB-Fc). Furthermore, compared with the control group, muscle fibers isolated from Mss51 knockout mice showed a higher rate of oxygen consumption, up-regulated expression of genes related to oxidative phosphorylation and fatty acid β-oxidation in muscle, and increased systemic glucose turnover and glycolysis rates, as well as enhanced systemic insulin sensitivity (70). This suggests that the antidiabetic effect of inhibition of MSTN may be achieved through MSS51 and enhancing mitochondrial oxidation.

In addition to its direct relationship with diabetes, MSTN is also involved in the occurrence and development of diabetes complications. In diabetic muscle atrophy rats, MSTN expression is increased in skeletal muscle and pulsed electromagnetic field intervention can decrease MSTN expression and increase the cross-sectional area of muscle fibers (71). Diabetic nephropathy is also a common microvascular disease in diabetes. In the kidney, MSTN is mainly expressed in the renal tubules and interstitium, and is colocalized with CD45^+^. In diabetic nephropathy patients, the expression level of MSTN in renal tubules is increased, and MSTN induced the release of ROS and up-regulation of NADPH oxidase in cells through the ERK pathway, thus aggravate the progression of tubule cell fibrosis (72).

### 4.3 MSTN in hypertension

MSTN have also been reported to be involved in another important clinical manifestation of metabolic syndrome: hypertension. Pucci et al. showed that individual with above-median serum MSTN concentrations had higher brachial diastolic blood pressure and higher carotid - femoral pulse velocity compared to controls (73). Similarly, compared to non-hypertensive healthy donors, the expression of MSTN was notably increased in structural cardiomyopathy patients (74). However, in the spontaneously hypertensive rat model, the expression of myostatin protein decreased in chronically hypertrophic myocardium and was significantly negatively correlated with left ventricular diastolic diameter/body weight ratio and left ventricular systolic diameter, but positively correlated with partial shortening of the middle wall (75). Although these parts reveal an association between MSTN and hypertension, further mechanisms need to be uncovered. In addition, whether the increase in MSTN levels in hypertensive patients is a primary or secondary increase also needs to be determined in the future.

### 4.4 MSTN-mediated tissue crosstalk in diseases

The expression of MSTN is not limited to skeletal muscle, it is also expressed in myocardium, adipose tissue, brain, kidney and circulating white blood cells (4, 72). Although current studies on MSTN focus on skeletal muscle, adipose tissere-derived MSTN is also essential for maintaining homeostasis. Adipose tissue is not only a storage of energy, but also secretes a series of proteins called adipokines that regulate the function of distal organs through endocrine processes (76). Recently, MSTN has also been identified as an adipokine. Steculorum et al. have shown that myostatin is secreted in brown adipose tissue and reduces local insulin sensitivity in the form of autocrine (77). In addition, MSTN also mediates the crosstalk between adipose tissue and muscle. Kong et al. have showed that loss of the transcription factor IRF4 in mouse brown adipose tissue (BATI4KO) reduced mitochondrial function and athletic ability in muscle (5). Furthermore, RNA-seq analysis showed upregulation of MSTN in adipose tissue of BATI4KO mice compared with control mice, while reducing circulating MSTN levels by neutralizing antibodies or soluble receptors restored exercise ability in BATI4KO mice (5). Moreover, overexpression of IRF4 in mouse brown adipose tissue reduces circulating MSTN levels, thereby increasing muscle athletic ability (5). This evidence reveals the role of MSTN as an adipokine in maintaining muscle homeostasis. However, whether adipose tissue-derived MSTN also affects the function of other organs needs to be further investigated in the future.

## 5 The inhibitor of MSTN

In view of the importance of MSTN in regulating muscle homeostasis, the inhibitors targeting the MSTN signaling pathway have been developed for clinical use to improve patients with sarcopenia and muscular dystrophy. Currently, a variety of compounds have been found to improve muscle function by inhibiting MSTN. A variety of MSTN-neutralizing antibodies have been used to inhibit MSTN, such as MYO-029 (78), SOD1 (79), AMG745 (80), Regn647 (81), GYM329 (82), which have been observed to improve muscle homeostasis by inhibiting MSTN levels. In addition, the development of drugs targeting the MSTN receptor is also one of the ideas to block the MSTN signaling pathway and several compounds are also being tested in animals or in clinical trials. Dumonceaux et al. shown that there was increased muscle mass in the mice once effectively down-regulated activin receptor IIb mRNA by intramuscular injection of adeno-associated virus (AAV) encoding a specific shRNA (83). Moreover, ACE-031 is a soluble ActRIIB that binds to circulating MSTN to inhibit its action (84). It has been shown to increase muscle mass in the interveners. Multiple studies have demonstrated that ACE-031 interventions increase muscle mass in subjects (85–87). In addition, a variety of natural compounds were observed to inhibit MSTN expression, including Epicatechin (88), Fructus Schisandrae (89), Sulforaphane (90) and Fructus Schisandrae (91). Here we summarize some inhibitors and part agonists of MSTN to use MSTN as a therapeutic target for diseases (**Table 1**).

**Table 1:** The inhibitors or agonists of MSTN.

Category	Compounds	Effects on MSTN	References
MSTN neutralizing antibodies	MYO-029	Inhibition	(78)
	SOD1	Inhibition	(79)
	AMG-745	Inhibition	(80)
	Regn647	Inhibition	(81)
	GYM-329	Inhibition	(82)
	Domagrozumab	Inhibition	(92)
	ATA-842	Inhibition	(93)
	SRK-015	Inhibition	(94)
Target ActRIIB	ACE-031	Inhibition	(84)
	ACE-2494	Inhibition	(95)
	ActRIIB-mFc	Inhibition	(96)
	RAP-031	Inhibition	(97)
	RAP-435	Inhibition	(97)
Natural compounds	Epicatechin	Inhibition	(88)
	Fructus Schisandrae	Inhibition	(89)
	Sulforaphane	Inhibition	(90)
	Fructus Schisandrae	Inhibition	(90)
Others	GASP-1	Inhibition	(98)
	MOTS-c	Inhibition	(99)
	Fenofibrate	Inhibition	(100)
	Dexamethasone	Upregulation	(101)
	Metformin	Upregulation	(102)
	Alcohol	Upregulation	(103)
	Cigarette smoke extract	Upregulation	(104)
	Angiotensin II	Upregulation	(105)
	AICAR	Upregulation	(106)

## 6 Conclusion and perspective

Although MSTN is well known for its role as a key protein that regulates muscle homeostasis and prevents excessive muscle growth, its role in regulating metabolic processes, especially in metabolic syndrome, is also attracting increasing attention. It is involved in the development of obesity, diabetes, lipid disorders and hypertension, while gene knockout or drug inhibition of MSTN can effectively reduce the symptoms of metabolic syndrome. However, the benefits of MSTN therapy in metabolic syndrome still need to be validated in clinical patients. What needs to be determined is whether the circulating MSTN mainly comes from muscle, adipose tissue or other tissues. In addition, the baseline level of MSTN in the cycle at rest needs to be determined. Furthermore, the molecular mechanism of MSTN therapy against metabolic syndrome also needs to be further explored in the future. Although there are still many questions to be addressed, anti-MSTN therapy could be a potential target for metabolic syndrome.

## Author contributions

MY the first draft of the manuscript. CBL, NJ, YL, HZ, CRL,YH, WC, LL, and LX, provided consultations on the preparation of the work. SL contributed to manuscript revision, read, and approved the submitted version.
